# The relationship between ambulatory arterial stiffness index and incident atrial fibrillation

**DOI:** 10.1002/clc.24299

**Published:** 2024-06-14

**Authors:** Christopher J. Boos, Aung Hein, Tom Wardill, Sadaf Diamondali, Su Wai, Peter O'Kane, Ahmed Khattab

**Affiliations:** ^1^ Department of Cardiology University Hospitals Dorset Dorset UK; ^2^ Faculty of Health & Social Sciences Bournemouth University Bournemouth UK

**Keywords:** AASI, ambulatory arterial stiffness index, ambulatory blood pressure, atrial fibrillation

## Abstract

**Background:**

The ambulatory arterial stiffness index (AASI) is an indirect measure of blood pressure variability and arterial stiffness which are atrial fibrillation (AF) risk factors. The relationship between AASI and AF development has not been previously investigated and was the primary aim of this study.

**Methods:**

This was an observational cohort study of adults (aged 18–85 years) in sinus rhythm, who underwent 24‐h ambulatory blood pressure monitoring (ABPM) for the diagnosis of hypertension or its control.

**Results:**

Eight hundred and twenty‐one patients (49% men) aged 58.7 ± 15.3 years were followed up for a median of 4.0 years (3317 patient‐years). In total, 75 patients (9.1%) developed ≥1 AF episode during follow‐up. The mean AASI was 0.46 ± 0.17 (median 0.46). AASI values (0.52 ± 0.16 vs. 0.45 ± 0.17; *p* < .001) and the proportion of AASI values above the median (65.3% vs. 48.4%; *p* = .005) were greater among the patients who developed AF versus those that did not respectively. AASI significantly correlated with age (*r* = .49; 95% confidence interval: 0.44–0.54: *p* < .001). On Kaplan–Meier analysis, higher baseline AASI by median, tertiles, and quartiles were all significantly associated with AF development (*X* ^2^: 10.13; *p* < .001). On Cox regression analyses, both a 1‐standard deviation increase and AASI > median were independent predictors of AF, but this relationship was no longer significant when age was included in the model.

**Conclusions:**

AASI is an independent predictor of AF development. However, this relationship becomes insignificant after adjustment for age which is higher correlated with AASI.

## 1 INTRODUCTION

The societal burden of atrial fibrillation (AF) is huge. It is by far the most common sustained tachyarrhythmia encountered in clinical practice and affects up to one in four adults >40 years and one in three adults aged >55 years during their lifetime.^1^, ^2^ The prevalence of AF continues to dramatically increase and is primarily related to an aging population and the increasing societal burden of adults with AF‐predisposing comorbidities (e.g., obesity, hypertension, heart failure, etc.).^3^

The clinical consequences of AF are potentially significant. AF adversely affects quality of life and it is one of the strongest independent risk factors for ischemic stroke and heart failure.^4^, ^5^ Consequently, there is a compelling clinical need to identify patients at increased AF risk to reduce its potential adverse clinical sequelae.

The ambulatory arterial stiffness index (AASI) has emerged as an increasingly appreciated marker of cardiovascular risk.^6^ Increasing AASI has been independently linked to an increased risk of major adverse cardiovascular and all‐cause mortality.^6^, ^7^ AASI is unique as an ambulatory cardiovascular risk marker as it is an indirect measure of both arterial stiffness^8^, ^9^ and blood pressure variability.^10^, ^11^ Increasing arterial stiffness is strongly associated with both new‐onset AF and its recurrence.^12^ Arterial stiffness leads to increased left ventricular afterload and associated hypertrophy, left atrial dilatation, neurohormonal activation and a pro‐inflammatory response that are all linked to AF development.^12^ Increased blood pressure variability has also been linked to incident AF and is thought to reflect autonomic imbalance and the alterations in ventricular afterload and arterial shear stress which again may active similar pathways to that of increased arterial stiffness.^13^, ^14^

Data from cohort studies have consistently shown a strong and independent link between increasing AASI and risk of ischemic stroke.^15^ In hypertensive diabetic patients, AASI is associated with impaired left atrial function which is independent of left ventricular diastolic dysfunction, supporting its pathophysiological links to AF development.^16^ Despite this, the relationship between AASI and incident AF has not been investigated. In this study, we hypothesized that increased AASI would be independently associated with incident AF among a group of adults in sinus rhythm.

## 2 METHODS

### 2.1 Study population and design

This was an observational study that was conducted at two adjacent (Poole and Royal Bournemouth) Hospitals. Our cohort consisted of adults aged 18–85 years who underwent 24‐h ambulatory blood pressure monitoring (ABPM) for the diagnosis of hypertension, or its control. Patients with previous organ transplantation, persistent/permanent AF, stage IV or V chronic kidney disease, pregnancy or with active cancer were excluded. Patients with severe aortic stenosis, aortic coarctation, active infection, or vasculitis were also excluded.

### 2.2 Twenty‐four‐hour ambulatory blood pressure and AASI measurement

All tests were done using an automatic ABPM device (Spacelab 90207, Spacelab Healthcare). An automated oscillometric cuff was placed on the nondominant arm. Blood pressure measurements were set to 30 min intervals throughout a 24‐h recording period. The nighttime period was defined as the hours of 22:01 to 06:00 h and the daytime period as 06:01 to 22:00 h. Patients were only included if they had a minimum of 10 daytime and 5 nighttime ambulatory blood pressure measures during the 24 h recording period as previously described.^17^, ^18^ Patients were advised not to use ABPM during periods of night shift work. The presence of sinus rhythm was confirmed on a 12‐lead ECG or cardiac device check before the ABPM being performed. The AASI was calculated, as previously defined,^19^ as 1‐minus the regression slope of the diastolic to systolic blood pressure over the 24 h recording period. The full data for all the 24‐h ABPMs were stored on TOMCAT (Philips CVIS Healthcare) reporting software.

### 2.3 Blood tests

Venous blood for the measurement of full blood count and renal function were performed in local National Health Service laboratories.

### 2.4 Outcome and AF diagnosis

The primary outcome was a diagnosis of AF. The diagnosis was based on AF confirmation on a 12‐lead ECG or from ambulatory ECG or cardiac rhythm strip of >30 s duration. All patient health records were examined for the duration of follow up. Clinical information, including for the diagnosis of AF and confirmation of patient deaths, were obtained using the Dorset Primary Care and NHS Electronic Patient Records.

### 2.5 Ethical approval

This study and its experimental protocol were approved by the Poole Hospital Clinical Research and Innovation Department and the West of Scotland Research Ethics Committee (REC reference: 20/WS/0097). As this was a registry cohort study the need for written informed consent was deemed not to be necessary by the ethics committee.

### 2.6 Statistical analysis

Statistical analyses were performed with SPSS 26.0 (SPSS) and GraphPad Prism version 6.07 for Windows (GraphPad Software). Identification of normality of continuous data was undertaken using data inspection and frequency histograms and the D'Agostino‐Pearson normality test. Continuous data were presented as mean ± standard deviations (SDs) and median (interquartile range) for normally distributed and non‐normally distributed data, respectively. Two group comparisons of continuous data were performed using an unpaired *t* test and Mann–Whitney *U* tests for normal and non‐normally distributed data, respectively. Categorical data were examined using Fisher's exact tests and chi‐squared tests with a relative risk (95% confidence intervals [CIs]) as appropriate. Correlations were examined using the Pearson and Spearman coefficients (95% CI) for normally and skewed data, respectively.

We performed Kaplan–Meier time‐to‐events analyses to examine the risk of incident AF based on higher categorical AASI values based on the median, tertiles and AASI quartiles. The independent association between a 1‐SD increase and higher (>median) AASI was undertaken using Cox regression with adjustment for patient age, sex, previous AF history, history of hypertension and 24‐h diastolic blood pressure with results reported as hazard ratios (HR; 95% CI). Sensitivity analyses were also performed to assess the robustness of the final model by examining the influence of categorical age (>median vs. ≤median) and separately with only the inclusion of patients excluding without a history of previous AF. A two‐sided *p* value of <.05 was considered significant for all comparisons.

### 2.7 Sample size and power calculation

This was performed using a proprietary sample‐size calculator (GraphPad StatMate version 2.00 for Windows). There have been previous studies that have examined the relationship between AASI using 24‐h ABPM and AF. However, in previously published study Matsumoto et al examined 769 older adults and identified a significant relationship between 24‐h (adjusted HR of 1.24 per 10 mmHg) and new‐onset AF which affected 10.8% of their cohort (83/769).^20^ Our sample size of >769 patients was based on this publication and assuming a similar HR for AASI and incident AF.

## 3 RESULTS

### 3.1 Baseline demographics

A total of 821 patients who were aged 58.7 ± 15.3 (range 18–85) years were included; 49% were men and 97% were Caucasian. Hypertension (63.8%) was the commonest cardiac risk factor with 51 (6.2%) patents identified as having a previous history of AF. Fifteen patients had a cardiac device (pacemaker, implantable cardiac defibrillator, or loop recorder [ILR]).

### 3.2 Relationship between AASI and patient characteristics

There was an average of 27.1 ± 4.7 24‐h ABPM readings per patient. The mean AASI was 0.46 ± 0.17 with a median of 0.46. Patients with an AASI above the median (*n* = 410) versus ≤median (*n* = 411) were significantly older (65.1 ± 12.9 vs. 52.4 ± 14.9 years; *p* < .001), more likely to have a history of hypertension (287/410 vs. 234/411; *p* < .001), previous stroke/TIA (43/410 vs. 20/411; *p* = .003) and were less likely to be men (183/410 vs. 215/411; *p* = .030).

### 3.3 Relationship between patient characteristics and AF

The median follow‐up was 4.0 (range: 1–6.4) years. During 3317 patients‐years of follow‐up, 75 patients (9.1%) developed one or more episodes of AF. AF patients were older, had greater body mass, were more likely to have a history of hypertension, diabetes mellitus, previous stroke, or heart failure compared with those without AF development (Table 1). Patients who developed AF were more likely to be treated with a beta‐blocker, diuretic, or alpha‐blocker and had a lower baseline estimated glomerular filtration rate and left ventricular ejection fraction.

**Table 1:** Baseline demographics and clinical characteristics of the total cohort and patients with and without new‐onset atrial fibrillation (AF).

Characteristic	Full cohort	No AF	AF	*p* Value
Number	821	746	75	
Men	398 (48.5%)	353 (47.3%)	45 (60.0%)	.040
Age, years	58.74 ± 15.29	57.8 ± 15.3	68.8 ± 11.34	<.001
Caucasian	794 (96.7%)	719 (96.40%)	75 (100%)	.163
Height, cm	168.9 ± 10.6	168.7 ± 10.5	171.2 ± 11.23	.055
Body mass, kg/m^2^	81.7 ± 19.31	81.2 ± 18.9	87.3 ± 22.8	.009
Body mass index, kg/m^2^	28.6 ± 5.96	28.5 ± 5.86	29.7 ± 6.79	.086
Hypertension	521 (63.5%)	465 (62.3%)	56 (74.7%)	.043
Ischemic heart disease	164 (20.0%)	144 (19.3%)	20 (26.7%)	.131
Diabetes mellitus	128 (15.6%)	107 (14.3%)	21 (28.0%)	.004
Previous history of AF	51 (6.2%)	33 (4.4%)	18 (24.0%)	<.001
Previous stroke or TIA	63 (7.67%)	51 (6.85%)	12 (16.0%)	.010
Heart failure	38 (4.14%)	27 (3.62%)	11 (14.7%)	<.001
Current smoker	151 (18.4%)	139 (18.6%)	11 (14.7%)	.277
Medication				
ACE‐I/ARB	440 (53.6%)	395 (52.9%)	45 (62.7%)	.275
Calcium channel blocker	293 (35.7%)	261 (35.0%)	32 (39.9%)	.206
Beta‐blockers	264 (32.2%)	226 (30.3%)	38 (50.7%)	<.001
Diuretics	149 (18.1%)	124 (16.6%)	25 (33.3%)	<.001
Alpha‐blockers	97 (11.8%)	82 (11.0%)	15 (20.0%)	.036
Statins	314 (38.4%)	281 (37.7%)	33 (44.0%)	.319
Aldosterone antagonists	35 (3.82%)	30 (4.02%)	5 (6.67%)	.241
Left ventricular ejection fraction, %	58.9 ± 7.54	59.3 ± 7.12	56.1 ± 10.1	<.001
Hemoglobin, g/L	139.2 ± 15.4	139.3 ± 15.3	138.1 ± 15.6	.547
White cell count	7.33 ± 2.13	7.32 ± 2.12	7.38 ± 2.25	.815
Estimated GFR, mL/min/1.73 m^2^	70.9 ± 16.5	70.7 ± 16.4	66.97 ± 19.3	.048

*Note*: Categorical data are presented as numbers (%); *p* values refer to the difference between the patients with and without AF development during follow‐up.

Abbreviations: ACE‐I angiotensin‐converting enzyme inhibitor; ARB, angiotensin II receptor blocker; GFR, glomerular filtration rate; TIA, transient ischemic attack.

### 3.4 AASI, ambulatory blood pressure, and AF

AASI was significantly higher and 24‐h diastolic blood pressure and heart rate were lower among the patients with incident AF versus those without. There was also a greater proportion of patients with a higher AASI (>median 0.46) versus lower AASI in the incident AF compared with the non‐AF groups (49/75 vs. 361/746; RR: 1.89; 95% CI: 1.20–3.0; *p* = .005). Daytime diastolic blood pressure, mean arterial pressure, and heart rate were lower among the AF versus non‐AF patients (Table 2). Nighttime systolic blood pressure was higher, and there was greater systolic, diastolic, and mean arterial blood pressure dipping among the AF versus non‐AF patients.

**Table 2:** Baseline demographics and 24‐h ambulatory blood pressure readings of the full cohort and those with and without new‐onset atrial fibrillation (AF).

Characteristic	Full cohort	No AF	New‐onset AF	*p* Value
24‐h ABPM readings				
Number of readings	27.1 ± 4.74	27.1 ± 4.79	26.5 ± 4.20	.296
Systolic blood pressure, mmHg	132.7 ± 15.3	132.4 ± 15.8	134.1 ± 15.2	.379
Diastolic blood pressure, mmHg	76.4 ± 10.1	76.8 ± 10.0	72.5 ± 10.3	<.001
Heart rate	7067 ± 11.4	70.9 ± 11.3	67.2 ± 11.8	.007
Mean arterial pressure, mmHg	95.8 ± 10.2	95.9 ± 10.2	94.3 ± 10.0	.183
Ambulatory arterial stiffness index	0.46 ± 0.17	0.45 ± 0.17	0.52 ± 0.16	<.001
Daytime ABPM readings				
Systolic blood pressure, mmHg	136.8 ± 16.0	136.7 ± 15.6	137.0 ± 15.7	.885
Diastolic blood pressure, mmHg	69.6 ± 10.6	80.1 ± 10.5	74.9 ± 10.8	<.0001
Mean arterial blood pressure, mmHg	99.1 ± 10.6	99.30 ± 10.60	96.7 ± 10.4	.045
Heart rate	73.1 ± 12.4	73.5 ± 12.4	69.0 ± 11.9	.003
Nighttime ABPM readings				
Systolic blood pressure, mmHg	123.0 ± 15.9	122.4 ± 18.5	128.7 ± 18.7	.005
Diastolic blood pressure, mmHg	69.0 ± 10.3	69.1 ± 10.2	68.5 ± 11.1	.641
Mean arterial blood pressure, mmHg	88.1 ± 11.5	87.9 ± 11.4	90.0 ± 12.0	.133
Heart rate	65.1 ± 10.9	65.3 ± 10.8	63.5 ± 12.3	.188
Systolic dipping, %	10.2 (4.51–15.57)	10.7 (4.94–15.97)	7.07 (1.2–12.6)	<.001
Diastolic dipping, %	13.6 (7.53–19.55)	13.9 (7.98–19.94)	10.6 (2.09–15.5)	<.001
Mean arterial pressure dipping, %	11.3 (5.60–16.86)	11.7 (5.99–17.27	7.45 (0.30–14.1)	<.001

*Note*: *p* values refer to the difference between the patients with and without AF development during follow‐up.

Abbreviation: ABPM, ambulatory blood pressure monitor.

### 3.5 Outcome analyses

**Figure 1:**
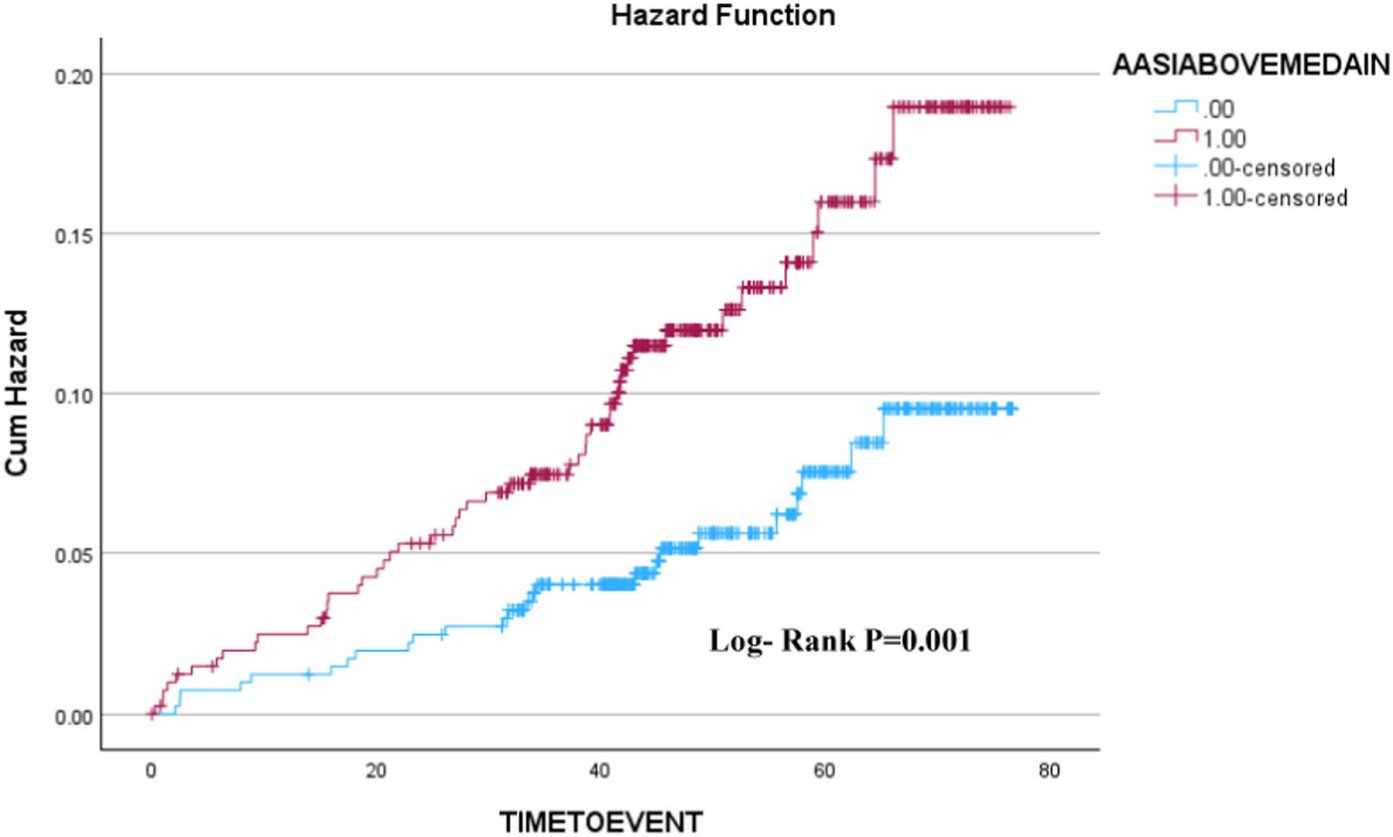
Kaplan–Meier graph showing AF event rate in patients with AASI above versus ≤median value of 0.46. AASI, ambulatory arterial stiffness index; AF, atrial fibrillation.

**Figure 2:**
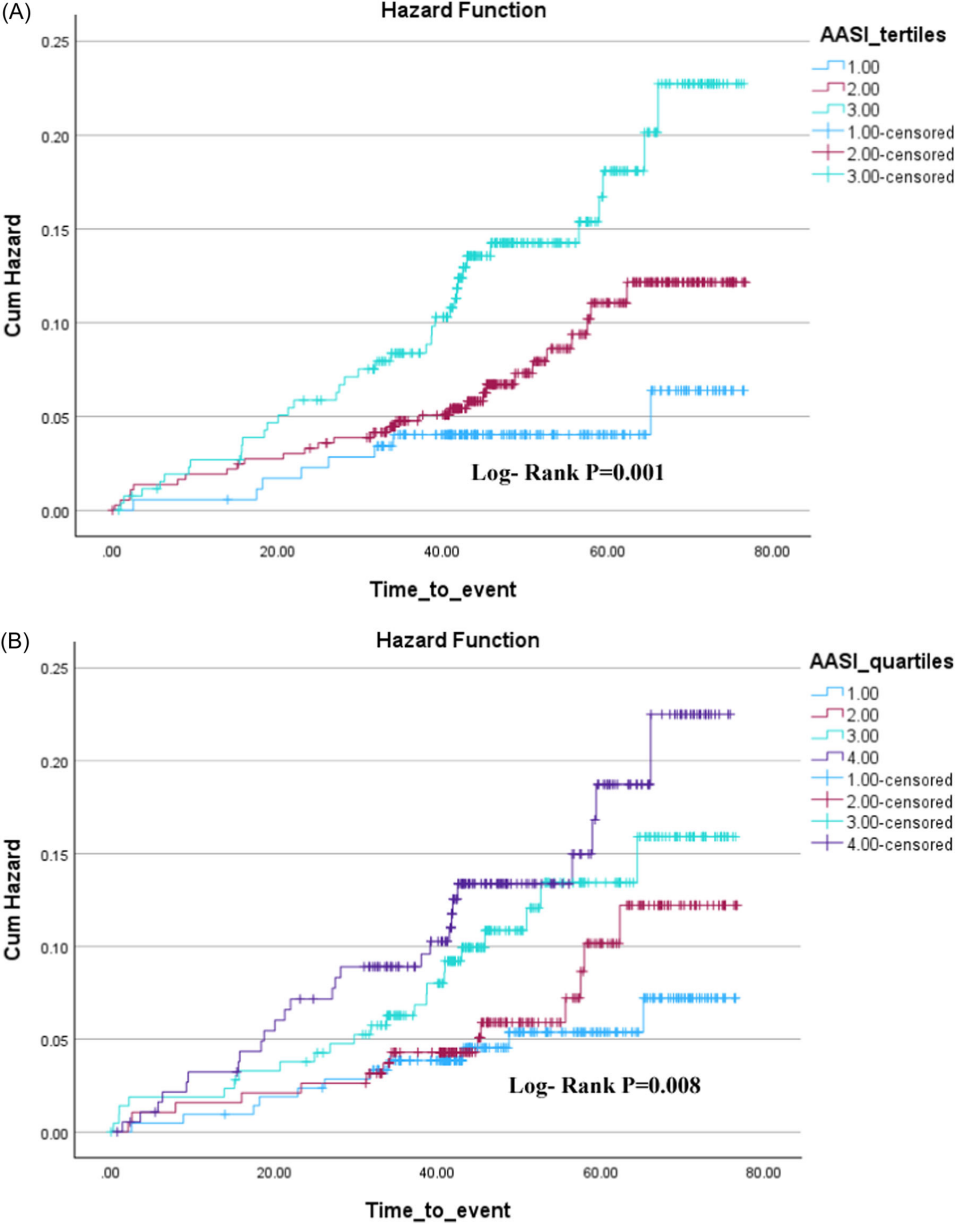
(A) Kaplan–Meier graph showing AF event rate in by AASI tertiles. (B) Kaplan–Meier graph showing AF event rate in by AASI quartiles. AASI, ambulatory arterial stiffness index; AF, atrial fibrillation.

On Kaplan–Meier analysis higher categorical AASI based on median (>0.46), tertiles (<0.33 1st, 0.34–0.53 2nd, >0.53 3rd) and AASI quartiles (<0.35 1st, 0.34–0.46 2nd, 0.47–0.57 3rd, >0.57 4th) AASI were all associated with a significantly higher risk of incident AF (Figures 1 and 2). On Cox regression analyses, a 1‐SD increase in AASI was a univariate predictor of future AF. On multivariate Cox regression analyses AASI (HR: 1.42; 95% CI: 1.11–1.82: *p* = .006), age, a previous history of AF, and male sex were independent predictors of incident AF (Table 3). However, following the inclusion of age into the model (along with sex, AF, history of hypertension, and diastolic blood pressure), the only independent predictors of AASI were age, male sex, previous AF, and no longer AASI (Table 3). Further Cox regression analyses using categorical AASI (above vs. ≤median) and similar covariate adjustments revealed similar results, with the independent prediction of higher AASI and AF being no longer significant after age (Table 3).

**Table 3:** Relationship between AASI and covariates to Incident atrial fibrillation (AF) development.

	Univariable model		Multivariable model 1		Multivariable model 2	
	Unadjusted hazard ratio (95% CI)	*p* Value	Adjusted hazard ratio (995% CI)	*p* Value	Adjusted Hazard ratio (95% CI)	*p* Value
1‐SD increase in AASI						
Male sex	1.60 (1.01–2.55)	.046	1.72 (1.07–2.74)	.024	1.71 (1.07–2.72)	.025
Hypertension	1.75 (1.03–2.98)	.039	1.50 (0.88–2.56)	.140	1.26 (0.73–2.16)	.404
Previous AF	5.44 (3.20–9.26)	<.001	4.19 (2.44–7.19)	<.001	3.74 (2.17–6.44)	<.001
1‐SD increase in AASI	1.55 (1.24–1.95)	<.001	1.42 (1.11–1.82)	.006	1.12 (0.85–1.49)	.421
Diastolic blood pressure (mmHg)	0.96 (0.94–0.98)	<.001	0.97 (0.95–0.99)	.018	0.99 (0.96–1.01)	.311
Age (years)	1.07 (1.04–1.09)	<.001			1.05 (1.03–1.08)	<.001
Categorical AASI (above vs. ≤median)						
Male sex	‐	‐	1.70 (1.06–2.70)	.027	1.69 (1.06–2.70)	.027
Hypertension	‐	‐	1.53 (0.89–2.61)	.121	1.27 (0.74–2.17)	.393
Previous AF	‐	‐	4.17 (2.42–7.16)	<.001	3.77 (2.19–6.50)	<.001
Higher AASI	2.15 (1.33–3.48)	.002	1.69 (1.03–2.79)	.040	1.06 (0.62–1.80)	.837
Diastolic blood pressure (mmHg)			0.97 (0.94–0.99)	.009	0.99 (0.96–1.01)	.285
Age (years)					1.06 (1.03–1.08)	<.001

*Note*: In model 1, the covariate adjustments were sex, history of hypertension, previous AF, AASI, and diastolic blood pressure; model 2 included model 1 plus additional adjustment for age.

Abbreviation: AASI, ambulatory arterial stiffness index.

Sensitivity Cox regression analyses were performed to examine the effects of higher AASI based on tertiles and quartiles on the outcome of AF. This did not mitigate the neutralizing effect of age. We also examined the impact of only including the 770 patients without a previous AF history for a 1‐SD increase in AASI and dichotomous AASI was similar with no significant and independent relationship between AASI and AF after age adjustment with the multivariable model.

## 4 DISCUSSION

This is the first study to investigate the relationship between AASI measured, using 24‐h ABPM, and AF. AASI was significantly higher among patients who subsequently developed AF versus those who remained in persistent sinus rhythm. On multivariable Cox regression analysis, increased and higher AASI were independent predictors of AF, but its significance was lost after adjustment for age, which was highly correlated with AASI.

AASI is emerging as a useful cardiovascular risk marker. Although the manual calculation of AASI can take several minutes, automated AASI results are now provided as part of 24‐h ABPM reporting software of several ABPM companies, including Spacelabs, used in this study. In our study, AASI was significantly higher among the patients who developed AF compared with those who did not. The strength of this relationship was enhanced given that the proportion of patients who developed AF was highest for each of the upper quantile, tertile, and quartile of AASI, with evidence of an ordinal effect. In a very recently published study of 8399 adults, it was shown that higher visit‐to‐visit systolic and diastolic blood pressure variability were both independently linked to incident AF.^14^ In another recent study of 769 adults Matsumoto et al. also demonstrated a significant relationship between 24‐h ABPM‐derived systolic and diastolic blood pressure variability and incident AF. In their study, the adjusted RR for incident AF per each 1‐SD increase in systolic blood pressure was 1.24 (95% CI: 1.11–1.37), and for diastolic blood pressure, was 1.30 (95% CI: 1.14–1.48). Furthermore, they observed that participants with the highest quartile of “both” systolic and diastolic blood pressure variability had the highest risk of incident AF, which is also supported by our data. AASI calculation itself reflects the linear relationship between diastolic and systolic blood pressure. Greater the variability in systolic blood pressure, diastolic blood pressure, or both would lead to a lower diastolic‐systolic regression slope and a greater AASI given its calculation as 1‐minus the diastolic‐systolic blood pressure regression slope.

ABPM is generally considered to be the gold standard test for the diagnosis of hypertension and has been shown to be a stronger prognostic indicator of both future AF and cardiovascular events compared to home or office blood pressure.^21^ Although AASI can be calculated using visit‐to‐visit blood pressure, its invention and prognostic value have been predominantly based using ABPM as used in our study. Moreover, unlike visit‐to‐visit blood pressure, ABPM addresses the influence of nocturnal blood pressure and its diastolic‐to‐systolic relationship and lessens the potential “white coat effects” of clinical visits on blood pressure indices. This is of major importance given that ABPM has been shown to be superior to both central and office blood pressure variability for AF prediction, supporting the premise for our study.^20^

In our study, we found that AASI was significantly higher amongst the patients with previous AF; this is a novel finding and strengthens our hypothesized AASI‐AF relationship. It has well‐established that previous AF is one of the strongest risk markers for future AF development which is enshrined in the ‘AF‐Begets AF’ doctrine.^22^ Among the covariates examined, we noted that age, previous AF and male sex were the only variables that were independently associated with incident AF in our fully adjusted Cox regression model. Indeed, age and male sex are two of the most consistently represented AF risk factors used in well‐validated AF prediction calculators.^23^, ^24^ The consistency and scale of the mitigating effect of age on the AASI‐AF relationship is interesting and raises the question as to whether age is in itself a confounder in the AASI relationship. Age has been consistently shown to strongly correlate with AASI, and this relationship was again supported by our data. Age is also strongly correlated and causally related to AF development.^25^ This makes the interpretation of our age‐adjusted Cox‐multivariable regression model quite challenging. It would appear that AASI acts as a mediator in the causal pathways between age and AF. The strong influence of age in the AASI‐AF relationship persisted even with the use of age as categorical variable and also the inclusion of only AF naïve patients.

This study has a number of limitations that need to be acknowledged. First, the sample size was modest but was powered based on a previous publication.^20^ With this sample size and 75 patients developing incident AF (9.1%) during our follow‐up period, the number of independent variables that we could interrogate in our regression model was limited. Second, the definition of AF was based on its confirmed presence on a 12‐lead ECG or cardiac rhythm strip conducted as part of routine clinical practice. Serial ECGs or ambulatory cardiac monitoring were not mandatory requirements for our study. Hence, the true incidence of new AF development post‐ABPM monitoring is likely to be higher than that reported. However, this is unlikely to have altered our results and as the vast majority of AF events were clinically driven (worsening symptoms and/or hospitalization triggering the need for an ECG or cardiac monitor) and important. We did not adjust for ethnicity as >97% of our total cohort and all of the incident AF cases were Caucasian. Finally, we did not examine the relationship between AASI to AF‐related adverse events (e.g., stroke, hospitalization, or heart failure), which is more clinically meaningful and will be the subject of future work.

## 5 CONCLUSIONS

In summary, in this study, we examined the relationship between 24‐h ABPM and incident AF. Both a 1‐SD increase and higher AASI were significantly associated with incident AF. This association was independent of sex, diastolic blood pressure, hypertension history, and history of AF. However, its significance was lost after adjusting for age. Further, larger studies are required to explore the relationship between AASI and adverse AF‐related clinical events.

## AUTHOR CONTRIBUTIONS

Christopher J. Boos designed the study, undertook the analyses, and wrote the first drafts manuscript. Tom Wardill, Sadaf Diamondali, Su Wai, and Aung Hein assisted with the data collection and assessment of study outcomes. Peter O'Kane and Ahmed Khattab helped with the writing and editing. All authors approved the final version of the manuscript.

## CONFLICT OF INTEREST STATEMENT

The authors declare no conflict of interest.

## Data Availability

The authors are willing to make the raw data available on a case‐by‐case request.

## References

[1] Staerk L , Wang B , Preis SR , et al. Lifetime risk of atrial fibrillation according to optimal, borderline, or elevated levels of risk factors: cohort study based on longitudinal data from the Framingham Heart Study. BMJ. 2018;361:k1453. 10.1136/bmj.k1453 29699974 PMC5917175

[2] Lloyd‐Jones DM , Wang TJ , Leip EP , et al. Lifetime risk for development of atrial fibrillation: the Framingham Heart Study. Circulation. 2004;110(9):1042‐1046. 10.1161/01.Cir.0000140263.20897.42 15313941

[3] Heidenreich PA , Trogdon JG , Khavjou OA , et al. Forecasting the future of cardiovascular disease in the United States: a policy statement from the American Heart Association. Circulation. 2011;123(8):933‐944. 10.1161/CIR.0b013e31820a55f5 21262990

[4] Freeman JV , Simon DN , Go AS , et al. Association between atrial fibrillation symptoms, quality of life, and patient outcomes: results from the Outcomes Registry for Better Informed Treatment of Atrial Fibrillation (ORBIT‐AF). Circ Cardiovasc Qual Outcomes. 2015;8(4):393‐402. 10.1161/circoutcomes.114.001303 26058720

[5] Benjamin EJ , Muntner P , Alonso A , et al. Heart Disease and Stroke Statistics‐2019 update: a report from the American Heart Association. Circulation. 2019;139(10):e56‐e528. 10.1161/cir.0000000000000659 30700139

[6] Aznaouridis K , Vlachopoulos C , Protogerou A , Stefanadis C . Ambulatory systolic‐diastolic pressure regression index as a predictor of clinical events: a meta‐analysis of longitudinal studies. Stroke. 2012;43(3):733‐739. 10.1161/STROKEAHA.111.636688 22282885

[7] Boos CJ , Thiri‐Toon L , Steadman CD , Khambekar S , Jordan A , Carpenter JP . The relationship between ambulatory arterial stiffness index and cardiovascular outcomes in women. Cardiol Res. 2021;12(3):161‐168. 10.14740/cr1189 34046110 PMC8139754

[8] Xu TY , Li Y , Fan WX , et al. Ambulatory (AASI), but not home (HASI), arterial stiffness index is associated with aortic pulse wave velocity. Hypertension Res. 2011;34(3):402‐403. 10.1038/hr.2010.248 21160480

[9] Heffernan KS . Peripheral augmentation index is associated with the ambulatory arterial stiffness index in patients with hypertension. Cardiol Res. 2011;2(5):218‐223. 10.4021/cr92w 28357009 PMC5358281

[10] Bahrainwala J , Patel A , Diaz KM , et al. Ambulatory Arterial Stiffness Index and circadian blood pressure variability. J Am Soc Hypertens. 2015;9(9):705‐710. 10.1016/j.jash.2015.07.001 26260424

[11] Lee HT , Lim YH , Kim BK , et al. The relationship between ambulatory arterial stiffness index and blood pressure variability in hypertensive patients. Korean Circ J. 2011;41(5):235‐240. 10.4070/kcj.2011.41.5.235 21731563 PMC3116100

[12] Lage JGB , Bortolotto AL , Scanavacca MI , Bortolotto LA , Darrieux FCC . Arterial stiffness and atrial fibrillation: a review. Clinics. 2022;77:100014. 10.1016/j.clinsp.2022.100014 35248986 PMC8903742

[13] Kikuya M , Asayama K , Ohkubo T . Blood pressure variability and arterial stiffness parameters derived from ambulatory blood pressure monitoring. Kardiol Pol. 2019;77(5):509‐514. 10.33963/kp.14845 31125026

[14] Kaze AD , Yuyun MF , Fonarow GC , Echouffo‐Tcheugui JB . Blood pressure variability and risk of atrial fibrillation in adults with type 2 diabetes. JACC Adv. 2023;2(4):100382. 10.1016/j.jacadv.2023.100382 37427148 PMC10328185

[15] Aznaouridis K , Vlachopoulos C , Masoura K , et al. Office blood pressure is a predictor of aortic elastic properties and urinary protein excretion in subjects with white coat hypertension. Int J Cardiol. 2016;203:98‐103. 10.1016/j.ijcard.2015.10.078 26498870

[16] Kalaycioglu E , Gokdeniz T , Aykan AC , et al. Ambulatory arterial stiffness index is associated with impaired left atrial mechanical functions in hypertensive diabetic patients: a speckle tracking study. Anatol J Cardiol. 2015;15(10):807‐813. 10.5152/akd.2014.5796 25592109 PMC5336966

[17] Verdecchia P , Angeli F , Mazzotta G , et al. Day‐night dip and early‐morning surge in blood pressure in hypertension: prognostic implications. Hypertension. 2012;60(1):34‐42. 10.1161/hypertensionaha.112.191858 22585951

[18] Presta V , Figliuzzi I , D'Agostino M , et al. Nocturnal blood pressure patterns and cardiovascular outcomes in patients with masked hypertension. J Clin Hypertens. 2018;20(9):1238‐1246. 10.1111/jch.13361 PMC8030919 30058135

[19] Dolan E , Thijs L , Li Y , et al. Ambulatory arterial stiffness index as a predictor of cardiovascular mortality in the Dublin Outcome Study. Hypertension. 2006;47(3):365‐370. 10.1161/01.HYP.0000200699.74641.c5 16432047

[20] Matsumoto K , Jin Z , Homma S , et al. Office, central and ambulatory blood pressure for predicting incident atrial fibrillation in older adults. J Hypertens. 2021;39(1):46‐52. 10.1097/hjh.0000000000002613 33031165

[21] Tikhonoff V , Kuznetsova T , Thijs L , et al. Ambulatory blood pressure and long‐term risk for atrial fibrillation. Heart. 2018;104(15):1263‐1270. 10.1136/heartjnl-2017-312488 29440183

[22] Wijffels MCEF , Kirchhof CJHJ , Dorland R , Allessie MA . Atrial fibrillation begets atrial fibrillation. A study in awake chronically instrumented goats. Circulation. 1995;92(7):1954‐1968. 10.1161/01.cir.92.7.1954 7671380

[23] Segan L , Canovas R , Nanayakkara S , et al. New‐onset atrial fibrillation prediction: the HARMS2‐AF risk score. Eur Heart J. 2023;44:3443‐3452. 10.1093/eurheartj/ehad375 37350480

[24] Orozco‐Beltran D , Quesada JA , Bertomeu‐Gonzalez V , et al. A new risk score to assess atrial fibrillation risk in hypertensive patients (ESCARVAL‐RISK Project). Sci Rep. 2020;10(1):4796. 10.1038/s41598-020-61437-w 32179807 PMC7075918

[25] Alonso A , Krijthe BP , Aspelund T , et al. Simple risk model predicts incidence of atrial fibrillation in a racially and geographically diverse population: the CHARGE‐AF consortium. J Am Heart Assoc. 2013;2(2):e000102. 10.1161/jaha.112.000102 23537808 PMC3647274

